# Remarks on the Treatment of Ophthalmia by the Nitrate of Silver

**Published:** 1825-02

**Authors:** T. H. Ridgway

**Affiliations:** late Surgeon to the third Battalion Rifle Brigade.


**Art. V.**—***Remarks on the Treatment of Ophthalmia by the Nitrate of Silver.*** By T. H. Ridgway, M.D. late Surgeon to the third Battalion Rifle Brigade.

In a late Number of your Journal, you reviewed some works and papers which have lately appeared on Ophthalmia; and I confess myself to have been much pleased with the acute and discriminating observations you have thought proper to bestow on them.

You have shrewdly remarked the coincidence between the plans of treatment pursued at Gibraltar and Chatham for the cure of ophthalmia, and have commented with much force on the conformity of opinion in the medical officers at these two stations. I can inform you that a similar method is employed in North America by Mr. Gibson, the intelligent surgeon of the 52d (Light Infantry); and I could furnish you with other coincidences of the same kind. My object, however, is not to surprise and puzzle you still further, but to endeavour to remove all difficulty, and to furnish a clue to unravel the perplexities in which this subject may otherwise be involved.

It will be seen, on reference, that the practice of introducing solutions of the nitrate of silver into the eye for the cure of ophthalmia, was communicated to the medical officers of the army in the Kent district, by a department order in the year 1812; which, for the sake of those who may not have access to the Book of Orders, I shall transcribe.*

***Extract from the Department Orders, dated Canterbury, August 17th,* 1812.**

**" A solution of ten grains of the nitrate of silver in an ounce of distilled water, has been found, in some of the regiments, an excellent application; a drop or two put into the eye every second day.**

**(Signed) " J. R. GRANT,**
**" Dep. Inspector of Hospitals."**

I may go further, and inform you that the prescription, on which this order was founded, was given by me to the deputy inspector of hospitals of the district, in writing, by his express desire, on his visit of inspection at Hythe, in Kent; and that this solution was adopted by me in consequence of observations made when in charge of the ophthalmic depot at Portsmouth in 1807, and afterwards in the Rifle Brigade, which had received the infection of ophthalmia from a regiment of the Egyptian

*** A list of no less than twenty regiments and medical stations are annexed to this order, with the signatures of the surgeons who received it, as is customary.**

expedition; and also that its use was transferred by me from ophthalmia to gonorrhœa.*

My regiment, the third battalion of the Rifle Brigade, was with the army in Paris in the year 1815. The Russian and Prussian armies had established their general hospital in the Hôtel Dieu, having turned out the patients for that purpose; and I observed that, amongst the Prussians, many of the soldiers were affected with an acute form of ophthalmia, which was protracted under the usual treatment, so that they filled one of the extensive wards of that magnificent structure. I sought out the medical officers, three or four of whom were in waiting, and to them I submitted the treatment by the solution of nitrate of silver, giving them the formula of ten grains to an ounce, with instructions, if I remember right, in writing.

Whilst in Paris, I instructed some medical officers of the light division in the use of the solution; and I personally urged its employment to Sir J. Grant, inspector-general of the army in France.

It will be immediately evident that the object of this communication is to assert a claim to a practice, which, as it has cost me much time, pains, and reflection, to project and mature, I do not feel disposed to resign quietly into the hands of others, who may have acquired but an imperfect knowledge of it,—who are unacquainted with the grounds on which it was adopted,—and are not aware of the extent to which it had been perfected before they had turned their attention to the subject. If I am asked wherefore I have not before published an account of this practice? I shall answer, without hesitation, that I had rather incur the imputation of negligence, than feel that I have deserved the charge of ignorantly and obtrusively forcing myself on the profession with crude views and undigested plans, to be corrected and matured by others.

Passing my life chiefly on active service and in the company of military men, I have felt little disposition, and had few opportunities, of employing myself in literary composition; but it will be found that I have not neglected to avail myself of the opportunities which have presented themselves of instructing those around me, and thus diffusing, in the most solid and useful manner, the information I may have accumulated. My practice was frequently brought for discussion before the Medical Society of the University of Dublin,† when my regiment was quartered

* The practice of treating gonorrhœa with nitrate of silver was begun by me about 1810, and was communicated officially to the Army Medical Board in 1817, by Mr. Freer, a medical officer stationed at Dover, who had served some time with my regiment in Flanders.

† Mr. Farley, of Charles-street, St. James's, chemist, informs me that the prescriptions for this solution, which have fallen into his hands, have all been made up for gentlemen from Ireland.

in that town; and it has been seen that I have not been wanting in communicating it to those who possessed the best means of promulgating it with effect.

From me, then, I may presume to assert, has emanated a practice which has already been found beneficial in both diseases, even in an imperfect shape, and which, on a full understanding of its merits, I cannot permit myself to doubt, will be found to deserve the favourable attention of the profession, for whose indulgence I hope one day to appeal.

Before I conclude, however, I must observe, that this practice has not been adopted empirically, but upon principles of general application: it will not, therefore, occasion surprise when I affirm, that, after it had been perfected to the highest pitch of which it was, perhaps, capable, in the practice of the regimental hospital of the third battalion Rifle Brigade before 1819,* it has in a great measure given way to, in my own practice, and been superseded by other remedies, which are afterwards to be demonstrated. This surely is not the time to "jump and cry out, ευρεκα!"

I have now, Gentlemen, to apologise for intruding on you with an explanation, which you will perceive has been in a manner forced upon me; and, as it is the first time, I trust the attempt will meet with indulgence from your liberality and disinterestedness.

* In the Pharmacopœia of the hospital of the third battalion Rifle Brigade, which was reduced in 1819, there are two formulæ for the solution of nitrate of silver,—one of ten grains, the other of half a drachm, to the ounce of water. The one of ten grains, however, was the first determined on by me, and was found the most generally useful. (Vide "a Treatise on Ophthalmia and Remittent Fever," pages 14 and 15.) The coincidence is perfect.

