# Errata in Dr. W. D. Dray's Paper, No. 312

**Published:** 1825-03

**Authors:** 


					InNo.312, ? 123, line 15, for " right" read "light."
? 124, line 19, for "^i?<t".read " si>ps*?."

				

